# Effect of obesity on constant workrate exercise in hyperinflated men with COPD

**DOI:** 10.1186/1471-2466-10-33

**Published:** 2010-05-30

**Authors:** Louis Laviolette, Francesco Sava, Denis E O'Donnell, Katherine A Webb, Alan L Hamilton, Steven Kesten, François Maltais

**Affiliations:** 1 Centre de recherche, Institut Universitaire de Cardiologie et de Pneumologie de Québec, Université Laval, Québec, Canada; 2 Respiratory Investigation Unit, Department of Medicine, Queen's University, Kingston, Ontario, Canada; 3 Boehringer Ingelheim (Canada) Limited, Burlington, Ontario, Canada; 4 Boehringer Ingelheim Corporation, Ingelheim, Germany

## Abstract

**Background:**

Chronic obstructive pulmonary disease (COPD) and a high body mass index (BMI) can both affect pulmonary volumes as well as exercise tolerance, but their combined effect on these outcomes is not well known. The aim of this study was to investigate the effects of increased BMI during constant workrate cycle ergometry in patients with COPD.

**Methods:**

Men with COPD and hyperinflation were divided according to World Health Organization BMI classification: 84 normal BMI (NBMI), 130 overweight (OW) and 64 obese (OB). Patients underwent spirometric and lung volumes assessment and an incremental cycling exercise test. This was followed by a constant workrate exercise test (CET) at 75% of peak capacity. Inspiratory capacity and Borg dyspnea scores were measured at baseline, during and at the end of CET.

**Results and discussion:**

FEV_1 _% predicted was not different across BMI classes. Total lung capacity and functional residual capacity were significantly lower in OB and OW compared to NBMI patients. Peak VO_2 _in L·min^-1 ^was significantly higher in OB and OW patients than in NBMI patients. CET time was not different across BMI classes (p = 0.11). Changes in lung volumes and dyspnea during CET were not different between BMI categories.

**Conclusions:**

OB and OW patients with COPD had a higher peak VO_2 _than their lean counterparts. Endurance time, dyspnea and changes in lung volumes during CET were similar between BMI categories.

## Background

COPD is characterized by reduced exercise tolerance [1], which is determined by a multitude of factors whose individual contribution may vary between patients[2]. Obesity is now an important issue in COPD: it is reported in 18% [3] to 54% [4] of patients with COPD and is associated with a better prognosis[5,6]. Both conditions, when taken individually, are characterized by physiological alterations in breathing mechanics along with functional limitations. The precise combined effects of COPD and obesity on such a multidimensional issue as exercise tolerance remain ambiguous. Clinical experience dictates that their combination would lead to a worsening of functional limitations and symptoms[7,8]. However, there are complex interactions between the two conditions that could mitigate a negative synergic effect on exercise performance.

On one hand, obesity is associated with an increased work of breathing [9], increased breathing resistive load [10] and worse exertional dyspnea [11]; combined with COPD, these factors would likely increase demands on an already stressed respiratory system and further reduce exercise tolerance. On the other hand, it is associated with decreased resting [12] and dynamic [13] lung volumes. Given the importance of dynamic hyperinflation in determining exercise tolerance in COPD [14], a reduction in lung volumes could lead to an increased exercise tolerance. Ora et al. [15] showed that because obese patients with COPD breathed at lower lung volumes during incremental cycling exercise, the perception of dyspnea at a given V_E _was reduced, allowing for greater peak VO_2 _power output during incremental cycling exercise.

The current study extends previous work by examining, for the first time, the impact of a broader range of increased body mass index (BMI, from overweight to severe obesity) on dyspnea and exercise endurance in a large population of hyperinflated men with COPD, whose airway obstruction varied from moderate to very severe. This information is relevant, given that overweight patients likely represent a substantial proportion of patients with an increased BMI. We also wish to evaluate the effects of overweight and obesity on the performance to constant workrate cycling exercise, an exercise testing modality that is gaining popularity to evaluate outcomes in COPD clinical trials[16].

The general aim of this study was to investigate the effects of increased BMI during constant workrate cycle ergometry. We hypothesized that, despite potentially worse exertional dyspnea and cost of breathing, increased BMI in association with COPD would be associated with preserved constant workrate exercise time because of lower resting hyperinflation, which provides a mechanical advantage on respiratory mechanics. The specific objectives of this study were: 1) to evaluate the effects of increased BMI on endurance time during constant workrate cycle ergometry; 2) to investigate the effect of increased BMI on operational lung volumes during exercise; 3) to investigate the effects of BMI on oxygen consumption, ventilation and symptoms intensity during constant workrate exercise.

## Methods

### Study subjects and design

This study is a pooled retrospective analysis of the baseline exercise data (prior to any pharmacological intervention) from two multicentre clinical trials examining the effects of tiotropium on exercise tolerance[17,18]. Local ethics committees approved the studies and all subjects signed written informed consent at the time of their assessment. For the purpose of the present study, only men were selected in order to minimize possible gender influence on lung volumes and exercise tolerance[19]. Three hundred and thirty five men with COPD were initially included in these two studies. Original inclusion criteria were: age 40 to 75 years, cigarette smoking > 10 pack-years, forced expiratory volume (FEV_1_) ≤ 65% predicted, and functional residual capacity (FRC) measured by body plethysmography ≥ 120% predicted. Patients with a history of asthma, allergic rhinitis or atopy were not eligible for the trial. Moreover, patients with any recognized contraindication to clinical exercise testing and patients who had participated in a rehabilitation program for COPD within 6 weeks prior to the screening visit were also not eligible. No patients participated in both original studies. For the present analysis, three inspiratory capacity (IC) data points or more (one at rest, one at submaximal exercise and one at end-exercise) during the constant workrate endurance test were necessary to examine changes in lung volume during exercise. Because IC maneuvers were performed at two-minute intervals, only patients with a constant workrate endurance time ≥ 4 minutes (n = 278) were included in the present analysis. Patients were classified according to BMI classification of the World Health Organization [20] into normal BMI (BMI 18.5 - 24.99 kg·m^-2^), overweight (25 - 29.99 kg·m^-2^) and obese (> 30 kg·m^-2^). Ideal weight was computed according to Wasserman et al.[21]

Baseline lung function, lung volumes and incremental exercise testing were performed during an initial screening visit. During a subsequent 2-3 week "run-in" phase to familiarize patients with testing procedures, they performed one constant workrate cycle exercise test (CET) on two occasions, without any pharmacological intervention. Results of the second test were used in the present analysis.

### Lung function evaluation

Spirometry and body plethysmography were performed according to the recognized standards[22-24]. FEV_1_, forced vital capacity (FVC), FEV_1_/FVC, FRC, total lung capacity (TLC) and residual volume (RV) values were then compared to the predicted values[22]. Predicted IC was calculated by subtracting the predicted values of FRC from the predicted value of TLC. Maximal voluntary ventilation (MVV) was calculated by multiplying FEV_1 _by 35[25]. Values for predicted DLCO were computed according to Cotes et al.[26]

### Exercise testing

The specific details of the incremental exercise test and the CET have previously been reported[17,18]. The incremental exercise test started with 3 min of unloaded pedaling after which workrate was increased in stepwise increments of 10 W·min^-1 ^from a starting point of 10 W. Subjects were instructed to cycle until symptom limitation. Peak work capacity (Wpeak) was defined as the highest workrate sustained for at least 30s. Reference values from Jones et al.[27] were used for peak exercise testing.

The CET started with 1 min of unloaded pedaling and workload was set at 75% of maximal capacity. Patients were instructed to cycle until symptom limitation at a pedaling rate of 50-70 rpm. Ventilatory and gas exchange parameters were collected breath-by-breath during exercise using commercially available cardiopulmonary exercise testing equipment. Values were recorded at rest and during the third 30-s period of each 2-min interval during exercise. End-exercise values were recorded as the average of the last 30 s of exercise. At rest, during each 2-min interval during exercise and at the end of exercise, subjects rated the intensity of dyspnea and leg discomfort using the modified Borg scale [28] and then performed an IC maneuver. The IC was used to track changes in operating lung volumes during exercise as previously described[29]. Oxygen pulse saturation (SpO_2_) was monitored during exercise.

### Statistical analysis

Results are shown as mean ± SD unless otherwise specified. Results are expressed as five time points: for each subject, data obtained at baseline, 25%, 50%, 75% and 100% of endurance time were used in the analysis. For continuous variables, BMI categories (normal BMI, overweight and obese) were compared first using ANOVAs. If statistical significance was reached, pairs were compared using Tukey-Kramer's HSD. The rate of hyperinflation during the constant workrate cycle exercise test (CET) was calculated by dividing the fall in IC by elapsed CET time. Statistical significance was set at p < 0.05.

## Results

### Patient's characteristics and static lung volumes

Table 1 shows patient characteristics according to BMI class. There were 84 male patients with normal BMI (BMI range: 18.62 - 24.96 kg/m^2^), 130 overweight patients (BMI range: 25.06 -29.86 kg/m^2^) and 64 obese patients (BMI range: 30.06 - 41.96 kg/m^2^). FEV_1 _and FVC were not significantly different between BMI classes. Resting lung volumes were significantly reduced in obese compared to normal weight patients.

**Table 1:** Baseline characteristics

	Normal BMI (NBMI), n = 84		Overweight (OW), n = 130		Obese (OB), n = 64		*p*		
							
							*NBMI*	*OW*	*NBMI*
	*Mean*	*(SD)*	*Mean*	*(SD)*	*Mean*	*(SD)*	*vs.*	*vs.*	*vs.*
							*OW*	*OB*	*OB*
Age, years	61.4	(7.2)	62.6	(6.4)	61.6	(6.6)	-	-	-
Tobacco exposure, Pack*years	52.3	(25.7)	59.0	(29.6)	55.8	(25.5)	-	-	-
BMI, kg*m^-2^	22.5	(1.7)	27.3	(1.5)	33.4	(3.1)	0.0001	0.0001	0.0001
Ideal weight, kg	77.6	(0.6)	77.9	(0.5)	76.9	(0.7)	-	-	-
FEV_1_, L	1.31	(0.39)	1.35	(0.45)	1.42	(0.49)	-	-	-
% predicted	40	(11)	42	(13)	44	(14)	-	-	-
FVC, L	3.09	(0.81)	3.04	(0.80)	2.89	(0.78)	-	-	-
% predicted	75	(18)	74	(17)	71	(17)	-	-	-
FEV_1_/FVC	42.9	(8.6)	44.5	(10.1)	48.9	(11.7)	-	0.05	0.01
TLC	8.38	(1.27)	8.04	(1.27)	7.79	(1.14)	-	-	0.05
% predicted	121	(17)	116	(17)	114	(14)	-	-	0.05
IC, L	2.46	(0.57)	2.51	(0.60)	2.50	(0.65)	-	-	-
% predicted	73	(15)	75	(17)	76	(18)	-	-	-
IC/TLC	30	(7)	31	(7)	32	(8)	-	-	-
FRC, L	6.06	(1.16)	5.66	(1.10)	5.40	(1.05)	0.05	-	0.001
% predicted	170	(31)	158	(29)	152	(27)	0.05	-	0.001
RV, L	5.02	(1.26)	4.63	(1.16)	4.53	(0.99)	-	-	0.05
% predicted	208	(50)	188	(50)	185	(46)	0.05	-	0.05
DLCO, ml*mmHg^-1^*min^-1^	15.4	(5.1)	16.2	(5.5)	19.2	(6.6)	-	0.01	0.001
% predicted	46	(22)	48	(24)	59	(27)	-	0.01	0.01
***Incremental exercise test***									
Peak VO_2_, L*min^-1^	1.204	(0.385)	1.375	(0.379)	1.550	(0.450)	0.01	0.05	0.0001
ml*kg^-1^*min^-1^	17.5	(5.3)	16.3	(4.0)	15.4	(4.4)	-	-	0.05
% predicted	50	(15)	57	(14)	65	(18)	0.01	0.01	0.0001
ml*kg_ideal weight_^-1^*min^-1^	15.5	(4.7)	17.5	(4.8)	20.2	(5.6)	0.01	0.01	0.0001
Peak V_E_, L*min^-1^	44.5	(13.8)	48.5	(13.5)	49.2	(14.3)	-	-	-
% MVV	100.3	(32.2)	108.6	(32.9)	108.0	(39.1)	-	-	-
Peak power, W	82.8	(28.1)	90.4	(26.7)	93.2	(35.1)	-	-	-
% predicted	45	(14)	50	(15)	52	(18)	-	-	0.05

Values are mean (SD), min - max. Definition of abbreviations: BMI: body mass index, FEV_1 _: forcedexpiratory volume in 1 second, FVC: Forced vital capacity, TLC: total lung capacity, IC: inspiratory capacity, FRC: functional residual capacity, RV: residual volume, DLCO: dilution capacity for carbon monoxide.

### Exercise tolerance

**Figure 1:**
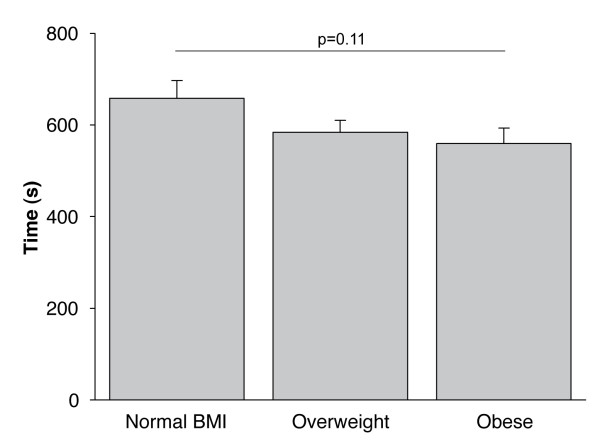
**Constant workrate cycle exercise performance (s) according to body mass index categories**. Values are mean ± SE.

Peak VO_2 _(L·min^-1^, % predicted and according to ideal body weight) was significantly increased, from normal BMI to obese patients. Figure 1 shows that CET time was not statistically different between the three groups (p = 0.11).

### Dynamic lung volumes

**Figure 2:**
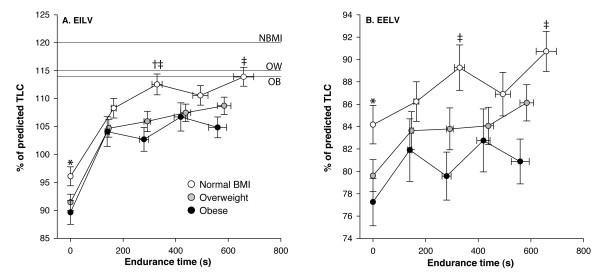
**Operating lung volumes during constant workrate cycle exercise expressed as a % of predicted total lung capacity according to body mass index categories**. Panel A shows end-inspiratory lung volume (EILV) and panel B, end-expiratory lung volume (EELV). Data points represent mean values at baseline, 25%, 50%, 75% and 100% of constant workrate endurance time. Values are mean ± SE. White filled circles are normal BMI, grey are overweight and black, obese patients. Horizontal lines represent total lung capacity for normal BMI (NBMI), overweight, (OW) and obese (OB) patients. * = p < 0.05; † = p < 0.005; ‡ = p < 0.01.

Figure 2 shows dynamic lung volumes during the CET expressed as a % of predicted TLC. Obesity significantly reduced end-expiratory lung volume (EELV) and end-inspiratory lung volume (EILV) at rest and at 50% and 100% of CET time. However, IC (% of predicted TLC), tidal volume (V_T_, % of predicted TLC), and inspiratory lung volume (IRV, % of predicted TLC) were not significantly different between BMI categories at any time points during the CET. The total decrease in IC during CET was 421 ± 409, 462 ± 389 and 380 ± 487 ml, for normal BMI, overweight and obese respectively (p = 0.46). The rate of fall in IC was not significantly different between BMI categories: -0.83 ± 0.90, -0.96 ± 0.80 and -0.81 ± 1.14 ml·s^-1^, for normal BMI, overweight and obese respectively (p = 0.52).

### Breathing pattern and VO_2_

**Figure 3:**
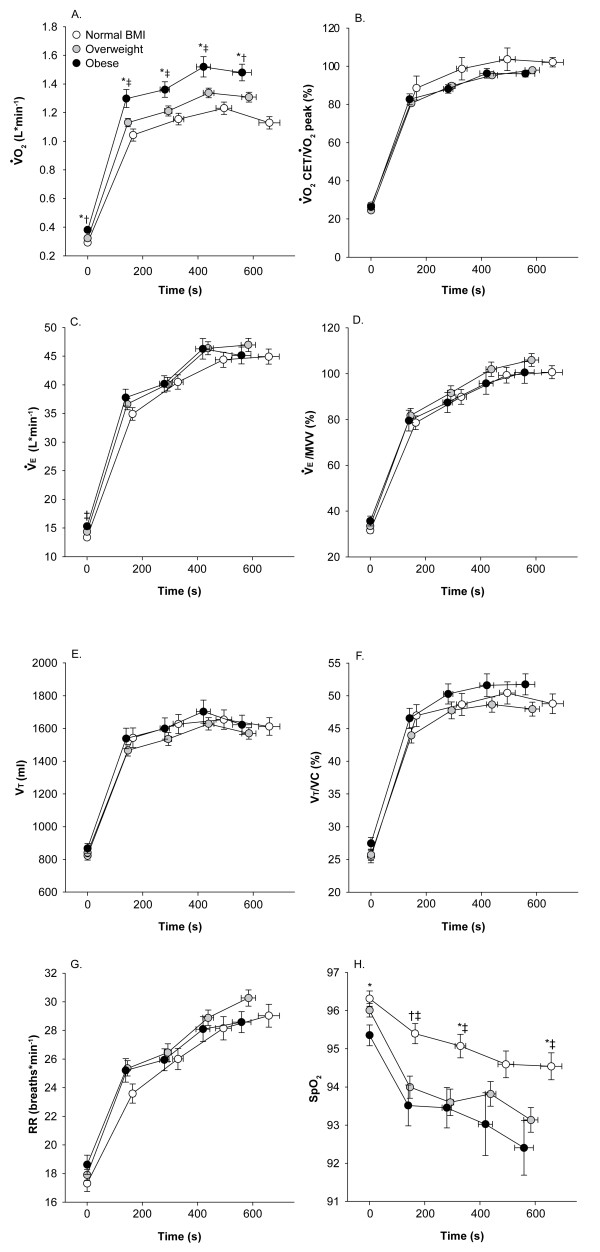
**Oxygen consumption (**VO_**2**_**, L·min**^**-1 **^**[panel A] and as a % of peak incremental values [panel B]) during constant workrate exercise (CET), ventilation (V**_**E**_**, L·min**^**-1 **^**[panel C] and as a % of MVV [panel D]), tidal volume (V**_**T**_**, ml [panel E) and as a % of vital capacity [panel F]), respiratory rate (RR, breaths·min**^**-1**^**) and O_2 _pulse saturation (SpO**_**2**_**) in relation to constant workrate endurance time (s)**. Values are mean ± SE. White filled circles are normal BMI, grey are overweight and black, obese patients. * = p < 0.0001; † = p < 0.01; ‡ = p < 0.001.

Figure 3 shows VO_2 _and breathing pattern during CET for all three BMI classes. VO_2 _(L&183;min^-1^) was higher as BMI increased at all time points during the CET, except at rest. When expressed in % of peak value attained during the incremental exercise test, VO_2 _was not significantly different between groups. Ventilation (V_E_), V_T _and respiratory rate (RR) were not significantly different across BMI classes during the CET. SpO_2 _showed significantly lower values during the CET in obese and overweight patients.

### Dyspnea and leg discomfort

**Figure 4:**
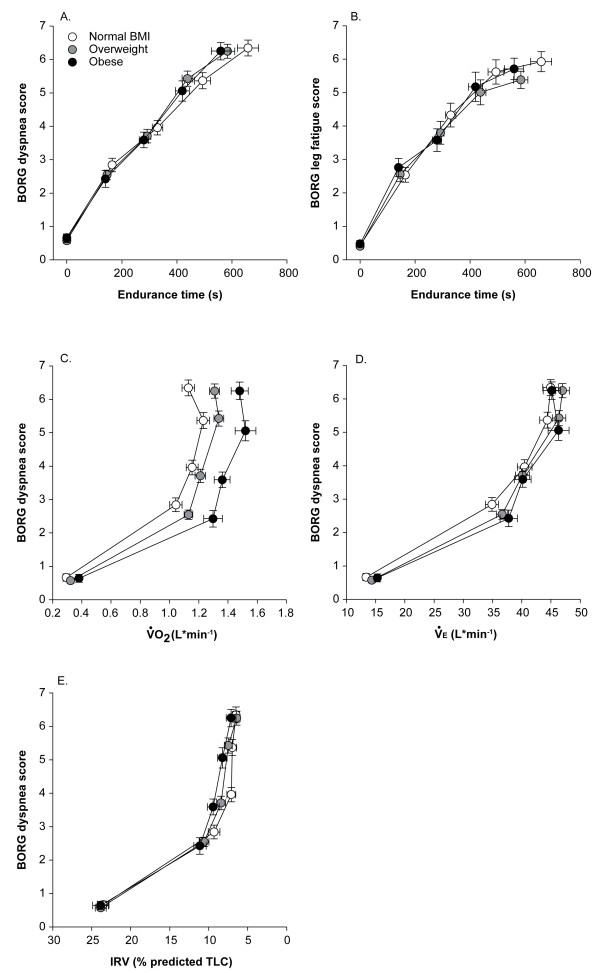
**Borg dyspnea (panel A) and leg fatigue (panel B) scores in relation to constant workrate endurance time, Borg dyspnea score in relation to oxygen consumption (VO**_**2**_**, L·min**^**-1**^**, panel C) ventilation (V**_**E**_**, L·min**^**-1**^**, panel D) and inspiratory reserve volume (IRV, % of predicted total lung capacity, panel E)**. Values are mean ± SE. White filled circles are normal BMI, grey are overweight and black, obese patients.

Time courses of dyspnea and leg discomfort during the CET (figure 4) were not significantly different across BMI classes. The dyspnea/VO_2 _ratio was shifted to the right with increasing BMI. As BMI increased, the steep rise in Borg dyspnea score occurred at higher VO_2 _(L·min^-1^). However, for a same value of V_E _(L·min^-1^) and IRV (% of predicted TLC), dyspnea was similar across the three BMI categories.

## Discussion

This study takes advantage of a unique exercise testing database in COPD to investigate the impact of obesity on constant workrate exercise tolerance and dynamic lung volumes in a large sample of male patients with COPD and resting lung hyperinflation. The main study results are as follows: 1) resting hyperinflation is reduced in the presence of increased BMI despite similar FEV_1_, 2) obesity is associated with higher incremental peak exercise VO_2 _while CET time was not statistically different across BMI categories, 3) absolute dynamic lung volumes (EELV, EILV) during the CET were reduced as BMI increased, but the overall rate of dynamic hyperinflation was similar across BMI categories, and 5) dyspnea ratings during the CET were not significantly different across BMI categories.

In this sample, obesity, compared to normal BMI, was associated with reduced resting hyperinflation and gas trapping. These results are consistent with observations in otherwise healthy subjects showing a significant reducing effect of increased BMI on static pulmonary volumes[12,13,30-32]. Our results are also similar to those of a previous mechanistic study investigating the effects of obesity in COPD on exertional dyspnea [15], indicating that the lung deflating effect of obesity is present in COPD, irrespective of the level of airway obstruction, which ranged from moderate to very severe. We submit that the added thoracic and abdominal weight in obese patients with COPD leads to decreased respiratory system compliance and to a reduction in static hyperinflation.

Our study shows that operating lung volumes were lower, but that tidal volume and inspiratory capacity were unchanged in obese patients with COPD. Interestingly, extending the results of Ora et al. [15] we confirmed that the rate and extent of dynamic pulmonary hyperinflation was not influenced by BMI across the range from overweight to severe obesity in a large population of patients with varying FEV_1_.

Dynamic lung volumes are subjected to multiple forces in patients with COPD with increased BMI. While resting hyperinflation was reduced in overweight and obese patients, the variations in EELV during exercise across BMI groups were not different. In men with normal lung function, the effects of obesity vary with the intensity of exercise, with lower intensities eliciting lung deflation and higher intensities, increases in EELV[31]. This increase in EELV toward the end of exercise was in part explained by the need to escape flow limitation as ventilation increased[31]. In the present study, flow-volume curves were not available to objectify flow limitation during exercise; however, given that the three groups behave almost identically in terms of dynamic hyperinflation irrespective of BMI, we submit that COPD was the main driving factor for dynamic lung volumes.

Increasing BMI over a wide range did not emerge as a disadvantage with respect to endurance time to tolerance at a standardized workload, when compared with their normal BMI counterparts. However, the design of constant workrate cycling exercise, while offering large sensitivity to interventions, may introduce some bias when comparing inter-patient performance. In our study, obese patients reached higher absolute and relative peak VO_2_. Therefore, they exercised at a higher absolute VO_2 _during CET. However, VO_2_. during the CET, expressed as a percentage of VO_2 _during the incremental test, was not significantly different between BMI categories Therefore, the relative intensity of the CET was uniform across BMI categories.

In theory, breathing at lower operating lung volumes should place the respiratory muscles (particularly the diaphragm) in a more favorable position to generate tension and therefore mitigate some of the negative mechanical and metabolic consequences of COPD during exercise. The net effect of combining higher BMI with COPD was not disadvantageous during cycle exercise (compared with normal BMI COPD): the change in dyspnea as a function of time or V_E _across the three BMI categories (figure 4) was similar. The V_E_/MVV relationships were also strikingly similar across the three BMI categories (figure 3). The fact that CET time was not decreased in obese patients can be easily understood when considering the absence of notable difference in the time courses of dyspnea and V_E _between BMI categories. At variance with our results, Ora and colleagues reported that dyspnea per unit V_E _was lower in obese compared to lean patients with COPD[33]. This difference between studies can be explained, in part, by the exercise protocol used (constant work rate in the present study versus incremental in Ora's study). Also, Ora's study was ideally suited to highlight the impact of obesity on the dyspnea/V_E _relationship because the differences in lung volumes between BMI categories were important and larger than in the present study.

### Limitations of the study

Because the present study involved a large sample size, a wide range of disease severity and was rigorously conducted in experienced research centers, we feel confident about the validity of the conclusions set forth. However, measurements made in this study did not allow us to investigate the mechanisms involved in dynamic hyperinflation. One limitation is that patients with COPD were not phenotyped as a part of this study. It might be argued that the proportion of emphysematous patients might have been greater in those with normal BMI. However, recent observations do not support this traditional theory. The correlation between BMI and the severity of emphysema is weak [34] and a recent sub-analysis of the NETT trial, in which computed tomography was performed to confirm emphysema, showed that the mean BMI of emphysematous patients was 24.6 ± 3.6, well within normal range and on the verge of overweight[35].

This study was conducted in a sample of men with FRC > 120% as an entry criteria. This requirement for hyperinflation could have attenuated the difference in exercise performance between BMI categories. Also, since patients were selected to have significant hyperinflation, the effects of obesity on COPD with less severe hyperinflation are unknown. Because of the potentially different mechanisms of hyperinflation between men and women [19], these results might not be applicable to obese women with COPD. Lastly, as BMI does not provide information on fat distribution, it would be of interest to investigate whether central versus peripheral obesity exert similarly influence on the exercise response.

### Conclusions

Increased BMI was associated with an increased peak incremental VO_2 _while endurance to constant workrate cycling exercise at the same relative intensity was similar across BMI categories. Static and dynamic lung volumes were reduced as BMI increased. Despite this, the rate of change in lung volumes during exercise was not modified in overweight and obese COPD. Exertional dyspnea was not significantly different across BMI categories. Overall, the effect of obesity in male patients with COPD and hyperinflation was not disadvantageous in terms of exercise endurance and exertional dyspnea during constant workrate cycling exercise.

## List of abbreviations

BMI: body mass index; CET: constant workrate cycle exercise test; COPD: chronic obstructive pulmonary disease; EELV: end-expiratory lung volume; EILV: end-inspiratory lung volume; FEV1: forced expiratory volume in 1 second; FRC: functional residual capacity; FVC: Forced vital capacity; IC: inspiratory capacity; IRV: inspiratory reserve volume; MVV: maximal voluntary ventilation; NBMI: normal BMI; OB: obese; OW: overweight; RR: respiratory rate; RV: residual volume; SD: standard deviation; SpO_2_: oxygen pulse saturation; TLC: total lung capacity; V_E_: ventilation; VO_2_: oxygen consumption; V_T_: tidal volume.

## Competing interests

L. Laviolette is a part-time consultant for Boehringer Ingelheim Canada. Dr Sava has no conflicts of interest to disclose. Dr O'Donnell has served on advisory boards for Boehringer Ingelheim, Pfizer, GSK and Roche, has received speaker's fees from Boehringer Ingelheim, Pfizer and GSK, and has received industry-sponsored research grants from AstraZeneca, Boehringer Ingelheim, GSK, Merck Frost Canada, Novartis and Pfizer. K. Webb has no conflicts of interest to disclose. Dr Hamilkton is an employee of Boehringer Ingelheim. Dr Kesten is a full time employee of Boehringer Ingelheim. Dr Maltais has no conflicts of interest to disclose.

The authors declare that they have no competing interests

## Authors' contributions

LL participated in the design of the study, performed the data analysis and drafted the manuscript. FS participated in the data analysis and helped draft the manuscript. DO and KW participated in the data analysis and extensively reviewed the manuscript. AH and SK participated in the original studies, provided the raw study data and reviewed the manuscript. FM conceived the study, participated in its design and data analysis and helped draft the manuscript. All authors read and approved the final manuscript.

## Pre-publication history

The pre-publication history for this paper can be accessed here:

http://www.biomedcentral.com/1471-2466/10/33/prepub
